# Congenital Disorders of Glycosphingolipid Biosynthesis: Ultrarare Severe Syndromes or Relatively Frequent Mild Neurocognitive Illnesses?

**DOI:** 10.3390/biomedicines14071506

**Published:** 2026-07-03

**Authors:** Linda Montavoci, Michele Dei Cas, Sara Penati, Marco Trinchera

**Affiliations:** 1 Department of Health Sciences, San Paolo Hospital, Università degli Studi di Milano, 20142 Milano, Italy or linda.montavoci@pharm.ox.ac.uk (L.M.); michele.deicas@unimi.it (M.D.C.); sara.penati@unimi.it (S.P.); 2 Department of Medicine and Surgery (DMC), University of Insubria, 21100 Varese, Italy

**Keywords:** ceramides, gangliosides, congenital disorders of glycosylation, epilepsy, intellectual disability

## Abstract

Glycosphingolipids (GSLs) are glycoconjugates in which a short and heterogeneous saccharide chain is attached to a lipid moiety called ceramide. Based on their sugar backbone, mammalian GSLs are primarily grouped into the ganglio-, lacto-/neolacto-, and globo-series. Sialic acid—containing GSLs are known as gangliosides. Complex ganglio-series gangliosides are particularly abundant in the brain, whereas simple ganglio-series gangliosides, as well as those belonging to other series or neutral GSLs, are less abundant and typical of non-neural tissues. Congenital disorders in the biosynthesis of the lipid moiety of sphingolipids (SLs) result from defects in enzymes and proteins involved in ceramide biosynthesis and transport. Congenital disorders in the biosynthesis of the sugar chain of GSLs specifically affect ganglio-series ganglioside biosynthesis and are caused by pathogenic variants in GM3 synthase (ST3GAL5) or GM2/GD2/asialo-GM2 synthase (B4GALNT1). Defective variants of the sialyltransferase ST3GAL3 and the galactosyltransferase B4GALT5 have been reported and proposed to impair GSL biosynthesis. The occurrence of these syndromes has provided new insights into the physiological and pathological roles of GSLs. Most of these disorders are associated with completely inactive enzyme variants, leading to severe neurological syndromes. Only a few cases highlighted variants that retained partial activity, resulting in milder phenotypes, which included non-syndromic intellectual disability. It is therefore conceivable that many undiagnosed patients, with mild neurological symptoms, may carry variants retaining residual enzyme activity, insufficient to ensure normal levels of brain GSLs. The purpose of this article is to encourage clinicians to look for additional GLS hereditary disorders associated with a milder phenotype. We also hope to boost future investigations by highlighting the most critical issues emerging from recent literature on SL and GSL biosynthesis and their related defects.

## 1. Introduction

**Figure 1:**
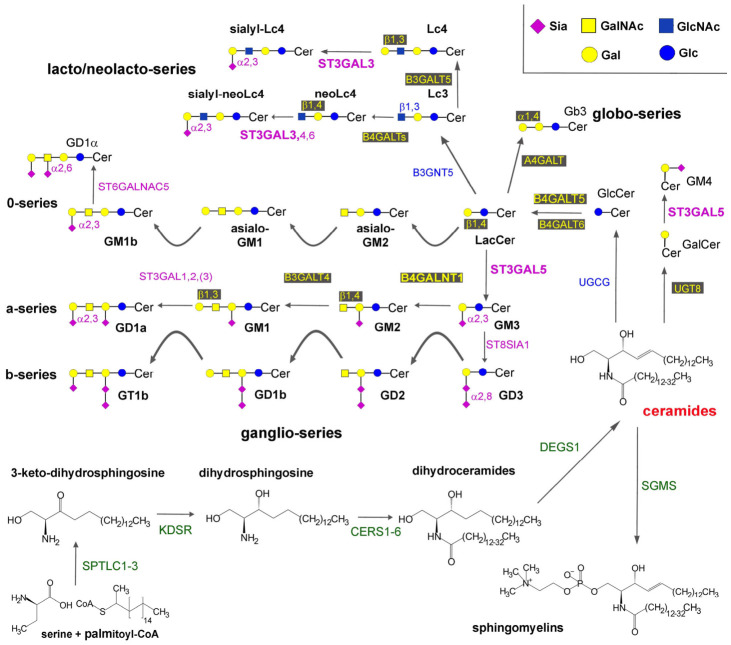
Main steps toward SL and GSL biosynthesis. Monosaccharides are depicted according to the current representation: Glc, glucose; Gal, galactose; GlcNAc, N-acetylglucosamine; GalNAc, N-acetylgalactosamine; Sia, sialic acid. Enzyme symbols are according to the HUGO nomenclature. UGT8, GalCer synthase; SPTLC, serine palmitoyl transferase; KDSR, 3-keto-dihydro-sphingosine reductase; CERS, ceramide synthase; DEGS, sphingolipid-4-desaturase; SGMS, sphingomyelin synthase; UGT8, GalCer synthase; UGCG, GlcCer synthase; B4GALT, β1,4-galactosyltransferase; A4GALT, α1,4-galactosyltransferase; B3GNT, β1,3-GlcNAc transferase; B4GALNT, β1,4-GalNAc transferase B3GALT, β1,3-galactosyltransferase; ST3GAL, galactoside-α2,3-sialyltransferase; ST8SIA, sialoside-α2,8-sialyltransferase; Lc3, lactotriaosylceramide; Lc4, lactotetraosylceramide; neoLc4, neolactotetraosylceramide; Gb3, globotriaosylceramide. Gangliosides are designed according to the current Svennerholm’s nomenclature.

The glycosphingolipids (GSLs) represent a class of glycoconjugates in which a short saccharide chain is attached to ceramide (Cer) and extends outward from the plasma membrane, facing the extracellular environment. GSLs are ubiquitously present across mammalian tissues; however, their amount and molecular profiles are highly tissue- and cell-specific. Both the Cer and sugar portions of GSLs exhibit considerable heterogeneity. Cer usually contains long-chain bases of 18 or 20 carbon atoms, which may also be hydroxylated. The fatty acids linked to these bases exhibit even greater diversity, most often spanning 14–24 carbon atoms but occasionally extending to 36, as in Ceramide Esterified Omega-Hydroxy Sphingosine (CerEOS), and they, too, may be unsaturated or hydroxylated. Beyond the structural diversity of Cer species, the sugar composition of the oligosaccharide chain itself is highly heterogeneous, giving rise to distinct subclasses (series) of GSLs that have been recognized for decades (Figure 1). Acidic GSLs contain sulfated or acidic sugars, notably sialic acids, whereas neutral GSLs lack these groups. The first sugar linked to Cer may be either galactose or glucose, forming galactosylceramide or glucosylceramide, respectively. Galactosylceramide can be further sulfated or sialylated, producing sulfatide or ganglioside GM4. Glucosylceramide can be extended by a galactose residue through a β1,4 linkage, forming lactosylceramide (LacCer)—the common precursor of the major GSL series. If another galactose residue is added to LacCer via an α1,4 linkage, the globo-series of GSLs is synthesized. The addition of an N-acetylglucosamine (GlcNAc), through a β1,3 linkage, initiates the lacto-/neolacto-series, whereas the addition of sialic acid or N-acetylgalactosamine (GalNAc) to LacCer marks the onset of the ganglio-series. Galactose, fucose, N-acetylhexosamines, and sialic acid contribute in various combinations to elongate and modify these glycan chains. The current understanding, as well as the remaining unsolved questions regarding the biosynthesis and biological roles of GSLs, has been recently reviewed and discussed in comprehensive articles [1,2,3].

Generally, complex polysialylated ganglio-series gangliosides, such as GD1a, GD1b, and GT1b, and even monosialylated ganglioside GM1, are defined as brain gangliosides, as they are particularly abundant exclusively in neural tissue. In contrast, simple ganglio-series gangliosides (e.g., GM3) and those of the lacto-, neolacto-, and globo-series, along with neutral GSLs, are mainly found in non-neural tissues, where their levels are much lower than those of ganglio-series gangliosides in the brain. Recent evidence indicates that the fine composition of the lipid moiety in certain GSLs may be altered in pathological conditions, sometimes even more markedly than the overall content of individual GSL species or classes. In the brains of patients affected by Parkinson’s disease, a reduction in the proportion of long-chain fatty acids within the lipid portion of several GSLs has been reported, representing a potential molecular signature of the disease [4].

A considerable number of studies, involving various cellular models, have investigated the function of neutral GSLs and gangliosides, revealing numerous potential associations with cancer as well as with many types of cell–cell and cell–ligand interactions [1,2]. Further functional insights have been obtained from knockout (KO) mice lacking one or more glycosyltransferases—the enzymes responsible for the biosynthesis of the different saccharide chains. Finally, the identification of human syndromes caused by impaired GSL biosynthesis has provided fundamental, but sometimes unexpected, insights into the physiological and pathological roles of these molecules. This article aims to stimulate future research by outlining the key issues emerging from recent literature on the growing group of disorders affecting sphingolipid (SL) and GSL biosynthesis. In particular, the working hypothesis is that this group of recessive metabolic disease determines severe clinical syndromes when enzyme activity is totally lost and they are extremely rare. Conversely, if the enzyme activity is partially maintained giving rise to milder phenotypes, they may be relatively more common but are poorly recognized so far. We want to encourage clinicians to look for additional hereditary disorders of GSL biosynthesis due to partially impaired enzyme activity and resulting in milder neurocognitive syndromes or even non syndromic intellectual disability.

## 2. Diseases Affecting the Biosynthesis of the Lipid Moiety of SLs/GSLs

To date, several congenital disorders of the lipid moiety of SL/GSL biosynthesis have been described. This group primarily involves Cer and sphingomyelin biosynthesis or transport and includes those related to pathogenic variants of serine palmitoyltransferase, like SPTLC1, SPTLC2, and SPTLSSA [5,6,7,8], 3-ketosphinganine reductase KDRS [9], Cer synthases CERS1, CERS2, and CERS3 [9,10], sphingolipid-4-desaturase DEGS1 [9,11,12,13,14], and Cer transfer protein CERT1 [15]. It is worth noting that a specific subset of Cers, known as “skin ceramides”, is localized exclusively in the stratum corneum of the skin, where they fulfill skin-specific functions [16]. Inborn errors in skin-Cer biosynthesis have also been reported [9] and impair these skin-specific functions. In all other cases, defects of SL biosynthesis give rise to neurological manifestations. Disease-causing variants in SPT subunits often produce enzymes that actively generate hydroxy-GSLs or lead to the accumulation of normal GSLs due to the loss of a regulatory site. These variants are primarily associated with peripheral neuropathy, often of late onset. However, some variants are responsible for a juvenile form of amyotrophic lateral sclerosis [6,7] or hereditary spastic paraplegia [5]. Defects in CERS enzymes are linked to myoclonic epilepsy and cognitive impairment, while DEGS1 defects result in hypomyelinating leukodystrophy, accompanied by varying degrees of intellectual disability. The severity of neurological and cognitive impairment correlates directly with the residual enzymatic activity of the variants [14]. Pathogenic CERT1 variants disrupt the regulated transfer of Cer from the endoplasmic reticulum to the trans-Golgi network, where a relevant part of sphingomyelin biosynthesis occurs. Phosphorylation-mediated inactivation of CERT1 is essential to balance sphingomyelin production. However, CERT1 variants resist this inactivation, causing a neurological syndrome characterized by speech and motor delays and intellectual disability, the severity of which increases with greater resistance to inactivation [15]. At present, there are no data correlating enzyme defect, SL pattern or amounts in the brain, and specific clinical symptoms. For instance, in DEGS1-associated leukodystrophy the impaired Cer/dihydro-Cer ratio is supposed to be pathogenic and DEGS1 was found to be a mitochondria-associated endoplasmic reticulum membrane-resident enzyme [12]. However, how these findings affect myelinization inducing particular neurologic symptoms is unknown. On the other side, patients carrying partially active DEGS1 variants present late onset and much less severe symptoms [11].

## 3. Diseases Affecting the Biosynthesis of the Sugar Chain of GSLs

Two disorders within the family of congenital disorders of glycosylation (CDG) affect ganglio-series ganglioside biosynthesis due to defective variants in GM3 synthase ST3GAL5 [17,18,19] and GM2/GD2 synthase B4GALNT1 [20,21]. ST3GAL5-CDG (also known as Amish infantile epileptic syndrome, GM3 synthase deficiency, or Salt and pepper syndrome) is a dramatic neuromotor disorder characterized by therapy-resistant epilepsy, severe cognitive and motor impairment, deafness and blindness. Notably, abnormalities could take months to manifest. These diseases are not detectable during pregnancy, at birth, or in the first weeks of life. Often, abilities acquired by newborns are progressively lost over time, with microcephaly typically absent. All variants subjected to biochemical characterization showed no detectable in vitro activity. It is noteworthy that ST3GAL5 KO mice, extensively studied for several years, exhibit cochlear deafness [18], a phenotype substantially milder than the human syndrome. In these animals, gangliosides of the 0 pathway (Figure 1) are supposed to compensate for the loss of typical brain gangliosides, while no data are available about brain GSL pattern in patients, including potential compensation mechanisms. Most importantly, recent studies have provided tools for studying ST3GAL5-CDG pathogenesis and potential therapeutic strategies [22,23].

B4GALNT1-CDG clinically corresponds to hereditary spastic paraplegia 26 (HSP26). Although less severe than ST3GAL5-CDG, it remains one of the complicated forms of HSP. In particular, it features progressive lower-limb weakness along with additional neurological symptoms, including cognitive and developmental impairment. The severity of the clinical picture correlates with the specific variants, many of which lack catalytic activity [20]. From a neuropathological perspective, B4GALNT1 KO mice resemble the human disease. Notably, one of the previously discussed SPT pathogenic variant that causes HSP90 and reduces ganglioside synthesis, has been shown to protect against disease progression in a mouse model of HSP11 [24]. No pathogenic mechanism is known at present able to explain such clinical evidence.

Moreover, two other disorders are supposed to be related to GSL biosynthesis. ST3GAL3-CDG, a rare but well-characterized epileptic encephalopathy, arises from defective variants in the sialyltransferase ST3GAL3 [25,26,27]. The current hypothesis posits that gangliosides are affected more than glycoproteins [18,19], although this remains unproven. The second disorder is based on a single case report which associates mild cognitive impairment, microcephaly, and early-onset bilateral cataracts with inactive B4GALT5 variants [28]. However, definitive proof of a genotype–phenotype relationship is lacking. Moreover, studies in KO mouse models suggest that B4GALT6 can compensate for B4GALT5 loss in brain function, raising the question: do these symptoms result from the inactive enzyme, or are KO mouse models not predictive of human disease?

ST3GAL3-CDG is most commonly reported as a severe developmental epileptic encephalopathy associated with complete loss of enzyme activity. However, a patient carrying a variant retaining measurable activity [26,27] presented only non-syndromic intellectual disability, without additional neurological symptoms. Notably, ST3GAL3 KO mice appear largely healthy and without neurological impairment, in stark contrast to the severe epileptic encephalopathy observed in humans. Although sialylation plays a critical role in the nervous system [29], previous and recent reports [30] suggest that the lack of a single ST3GAL enzyme does not severely impair the human nervous system, owing to compensatory activity from other isoforms.

The structure of B4GALNT1 suggests a membrane-binding domain; this is typically observed in glycosyltransferases acting on membrane-embedded substrates, such as GSLs with short sugar chains [31]. Indeed, glucosylceramide synthase (UGCG), lactosylceramide synthases (B4GALT5/6), and GM3 synthase (ST3GAL5) are considered specific to GSL biosynthesis, due to their proximity to the lipid bilayer.

On the other hand, other galactosyl- and sialyl- transferases act on both glycoproteins and GSLs. This occurs because GSL substrates bear sugar chains long enough to protrude into the aqueous environment. Surprisingly, a recent report indicated that ST3GAL5 sialylates the glycoprotein CD177 on neutrophils [32]. Despite the reported preference of ST3GAL3 for GSLs [27], specific brain glycoproteins may also serve as relevant substrates for this enzyme, and such sialylated glycoproteins could potentially contribute to disease pathogenesis.

## 4. Congenital Disorders of Glycosylation: Glycoproteins and GSLs

Numerous CDGs have been described to date, most of which are syndromic in nature. Also, those affecting glycoprotein biosynthesis involve extra-neural tissues, especially in the most severe forms [33,34]. Conversely, disorders of GSL biosynthesis typically lack well-defined extra-neural involvement, and, in some cases, have been linked only to intellectual disability without neurological symptoms [27]. Complete loss of enzyme activity correlates with the most severe phenotypes, including neurological symptoms such as developmental delay, intellectual disability, seizures, and occasionally microcephaly. Equally, retention of residual catalytic activity results in milder symptoms, supporting the hypothesis that reduced GSL glycosyltransferase activity may underlie several currently unexplained (“orphan”) cases of non-syndromic intellectual disability.

Although glycosylation is ubiquitously necessary in many cell types, brain cells appear increasingly sensitive to any alteration in this process [35]. In other tissues, general reductions in glycoprotein or GSL content can often be compensated for by individual molecules. However, in the nervous system, the loss of glycosylation on a single protein or GSL can be pathogenic. Data on ganglioside profiles, observed in different brain regions from individuals with various neurological alterations, suggest that brain cells are highly sensitive to subtle changes in ganglioside composition [36,37]. At present, information concerning substrate specificity is enough for distinguishing glycoprotein and GSL dedicated enzymes at the initial steps of the glycosylation process. By contrast, whether elongation and termination of the saccharide chains of glycoprotein and GSLs occur through distinct or common enzymes, is not yet known. Scientific data, signatures, and mechanistic hypotheses are based on the distribution, abundance, and roles of the major brain ganglioside. A recent study showed that microglia–neuron interactions are essential for the degradative pathway of complex brain gangliosides, specifically for the conversion of GM2 into GM3. Normally present in trace amounts, GM2 accumulates if microglial cells fail to supply neurons with the β-subunit of β-hexosaminidase. This accumulation triggers lectin-mediated macrophage activation, leading to neurodegeneration [38]. Lysosomal GM2 buildup not only disrupts lysosomes—where ganglioside degradation occurs—but also alters plasma membrane composition in terms of glycoproteins and glycolipids, ultimately driving synaptic dysfunction [39]. In the peripheral nervous system, antibodies targeting gangliosides are frequently associated with Guillain–Barré syndrome. While antibodies against major gangliosides, such as GM1 and GQ1b (and their complexes), play a prominent role, antibodies against minor gangliosides, like GM1b, can also trigger the syndrome [40,41]. In this light, the pathophysiology of autism spectrum disorders has recently been linked to disruptions in the glycosylation homeostasis of both glycoproteins and gangliosides, driven by genetic variants, epigenetic dysregulation, and environmental factors [42]. Accordingly, proper glycosylation homeostasis has been proposed as essential for cognitive and emotional well-being [43].

## 5. Conclusions

Insights into the in vivo roles of GSLs, particularly gangliosides, have historically been derived from KO mouse models [2,18,19]. Although such investigations provided several important data points, it is now evident that KO mouse models fail to capture the complexity of the human brain and cannot reliably predict clinical disease features, as exemplified by ST3GAL5- and ST3GAL3-CDGs (see above). On the other hand, the physiological role of GSLs in the human nervous system could not be fully rearranged in the KO mouse models. The human nervous system likely lacks a uniform glycome, as distinct brain regions and cell types within each region may possess unique glycomes. Some GSLs appear minor in whole-brain analyses; they might play critical and irreplaceable roles in specific cell types or regions. Advances in mass spectrometry applied to post-mortem samples, organoids, and cultured neural cells offer promising tools for investigating these aspects. Moreover, these techniques have already proven effective for measuring variant enzyme activity in vitro [44,45] and determining substrate preferences—an aspect of particular relevance given evidence of unpredictable substrate specificities [32,46].

Among human syndromes caused by recessive defects in GSL biosynthesis, most reported cases involve completely inactive variants. Conversely, a small number of cases harbor variants with detectable residual activity (DEGS1, ST3GAL3, B4GALNT1), which are associated with milder phenotypes, including non-syndromic intellectual disability. The putative B4GALT5-associated disease is relatively mild compared to other CDGs, consistent with compensatory activity from B4GALT6, which is active but insufficient to maintain the required brain GSL levels. It is plausible that many undiagnosed patients with mild neurological symptoms preserve partially defective variants retaining residual activity. In addition, the same enzymes may cause severe syndromes when fully inactive. This perspective also highlights several areas that warrant further investigation: (1) Could patients with partially active ST3GAL5 present only hearing loss and mild cognitive impairment? (2) Do carriers of partially active ST3GAL3 manifest solely intellectual disability? (3) Might other enzyme variants produce mild neurological symptoms when partially active, but be incompatible with life when completely inactive? We invite clinicians encountering patients, particularly children, presenting with mild cognitive disability, attention deficit/hyperactivity symptoms, epileptic episodes, borderline occipital-frontal head circumference, hearing and/or visual impairment, or other minor neurologic symptoms of potential congenital origin, to take into account the possibility of a GSL-CDG. Plasma GSL profiles, obtained through mass spectrometry–based profiling, could help screen undiagnosed patients and guide targeted exome sequencing.

## Data Availability

No new data were created or analyzed in this study.

## Abbreviations

The following abbreviations are used in this manuscript:

B4GALNT	β1,4-GalNAc transferase
B4GALT	β1,4-Galactosytransferase
CDG	Congenital disorder of glycosylation
Cer	Ceramide
CerEOS	Ceramide Ester linked -Omega-hydroxy Sphingosine
CERS	Ceramide synthase
CERT	Ceramide transfer protein
DEGS	sphingolipid-4-desaturase
GD	disialoganglioside
GM	monosialoganglioside
GQ	tetrasialoganglioside
GT	trisialoganglioside
GSL	Glycosphingolipid
KDSR	3-ketosphinganine reductase
LacCer	Lactosylceramide
SL	Sphingolipid
SPTLC	Serine palmitoyltransferase
ST3GAL	Galactoside α2,3-sialyltransferase
